# The View from Afar: Satellite-Derived Estimates of Global PM_2.5_

**DOI:** 10.1289/ehp.123-A43

**Published:** 2015-02-01

**Authors:** Lindsey Konkel

**Affiliations:** Lindsey Konkel is a Worcester, MA–based journalist who reports on science, health, and the environment.

More than 3 million people died prematurely in 2010 due to ambient exposure to fine particulate matter (PM_2.5_), according to estimates from the Global Burden of Disease Study.^1^ Although air pollution measurements taken from ground-level monitors can help inform such estimates, a paucity of monitoring stations outside of North America and Western Europe make it difficult to compare levels and trends in PM_2.5_ and their health effects around the world.^2^ Fortunately, satellite data provide a way of filling in data gaps for areas with no ground-based monitoring. In this issue of *EHP*, a team of researchers report their satellite-derived estimates of global exposure trends to PM_2.5_ over 15 years.^3^

“We found notable trends of increasing PM_2.5_ in South and East Asia, where billions of people live. Meanwhile, parts of North America are getting cleaner,” says study author Randall Martin, an atmospheric scientist at Dalhousie University in Halifax, Nova Scotia.

**Figure:**
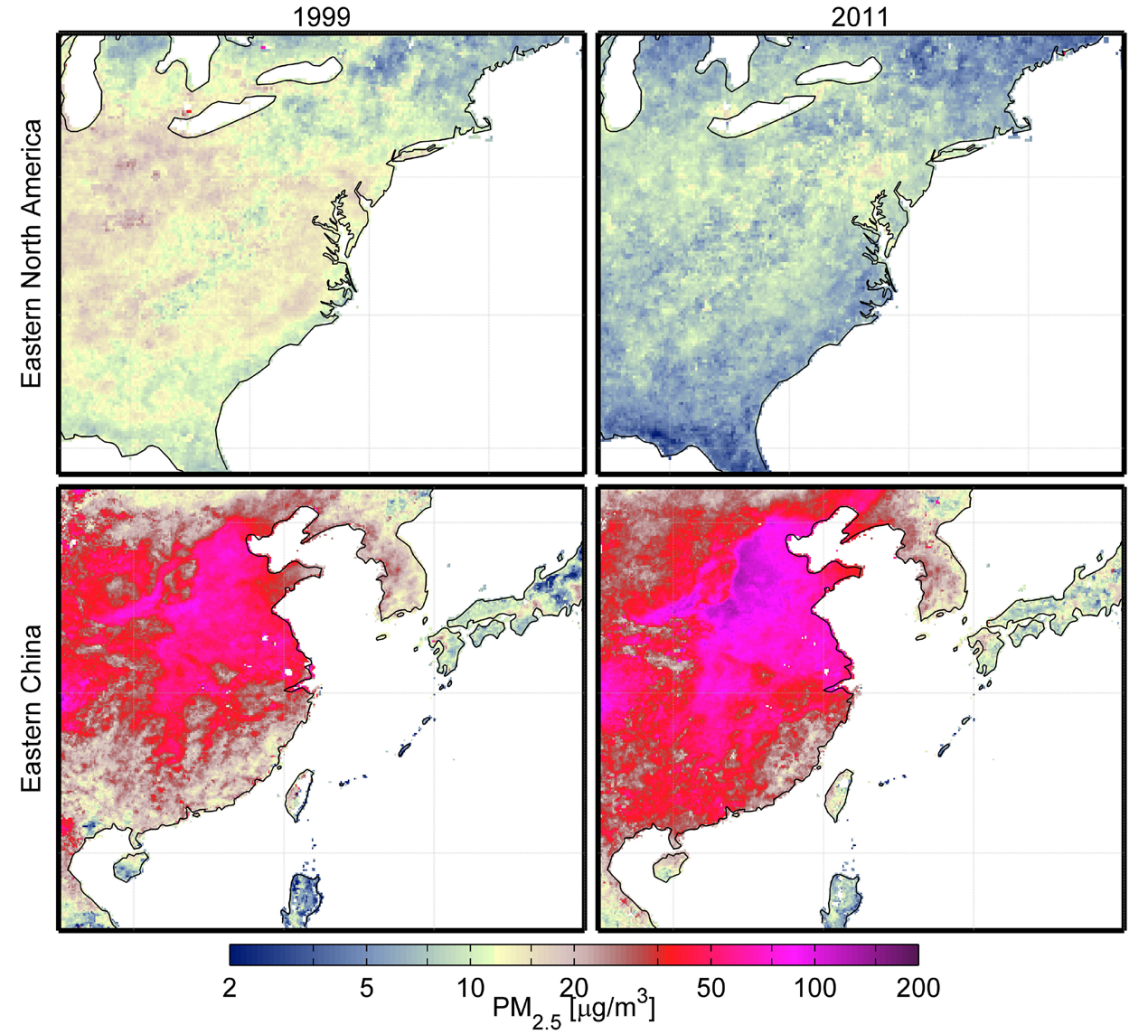
Satellite-derived estimates show PM_2.5_ pollution improving in some regions (top) and worsening in others (bottom) over a 15-year period. Source: van Donkelaar et al. (2015)^3^

Satellite sensors don’t measure PM_2.5_ directly. Instead, they assess how particles in the air, including PM_2.5_, scatter sunlight as it passes through the atmosphere. “In a sense, the satellites we use are little more than extremely well calibrated cameras that take pictures of the earth below. When aerosol particles are present, these pictures begin to look a little hazy,” explains first author Aaron van Donkelaar, also an atmospheric scientist at Dalhousie University. The extent to which aerosols scatter the light is called the aerosol optical depth (AOD).

The researchers used AOD data from the National Aeronautics and Space Administration to estimate ground-level PM_2.5_ at a spatial resolution of approximately 10 km × 10 km. Although some regions experienced a decrease in PM_2.5_ over the period 1998–2012, the global population-weighted average increased by an estimated 2.1% per year. Rising levels of air pollution in developing regions in South and East Asia largely drove the upward trend.^3^

After adjusting for population changes, the researchers estimated that the proportion of people in South and East Asia exposed to PM_2.5_ at levels exceeding the World Health Organization (WHO) interim target of 35 µg/m^3^ rose from 51% in 1998–2000 to 70% in 2010–2012. In contrast, the proportion of North Americans exposed to PM_2.5_ at levels above the WHO air quality guideline of 10 µg/m^3^ fell from 62% in 1998–2000 to 19% in 2010–2012.^4^ Where ground-level PM_2.5_ data were available, the researchers found a significant association with satellite-based estimates, though satellite-derived PM_2.5_ estimates tended to be slightly lower than ground-level readings.^3^

“Satellite-based estimates reported here will enable researchers to design and conduct large epidemiological studies in low- and middle-income countries that lack the extensive ground monitoring networks found in higher income countries,” says Aaron Cohen, an epidemiologist at the Health Effects Institute in Boston. He was not involved in the current study.

As satellite-derived PM_2.5_ estimates have become available, groups such as the Global Burden of Disease Study^1^ and the WHO^5^ have begun to use them as the basis for estimates of the global burden of disease. The current study builds upon a previous analysis by these authors, which estimated global PM_2.5_ levels from 2001 through 2006.^6^

Collecting data over an even longer period will allow researchers to talk with more confidence about trends in air pollution levels and link those trends to changes in health burden, according to Cohen. Researchers and policy makers can also track future progress as developing countries such as China begin to implement more stringent environmental regulations, he says.

Although the data set of 10 km × 10 km resolution may be useful for evaluating large global trends, it is not ideal for estimating associations between air pollution and health outcomes in individuals because there is too much heterogeneity in PM_2.5_ concentrations within such an area at any given point in time. Some air pollution estimates using satellite data at a resolution of 2.5 km × 2.5 km have been made in U.S. cities including Cleveland, Ohio, to assess more localized health risks from air pollution exposure.^7^^,^^8^ “In the future, we hope to infer global PM_2.5_ data at a higher spatial resolution and to investigate other air pollution compounds, such as nitrogen dioxide, using satellite data,” says van Donkelaar.
